# Substance-Related Problems in Adolescents with ADHD-Diagnoses: The Importance of Self-Reported Conduct Problems

**DOI:** 10.1177/10870547221105063

**Published:** 2022-06-25

**Authors:** Ove Heradstveit, Kristin Gärtner Askeland, Tormod Bøe, Astri Johansen Lundervold, Irene Bircow Elgen, Jens Christoffer Skogen, Mads Uffe Pedersen, Mari Hysing

**Affiliations:** 1 NORCE Norwegian Research Centre, Bergen, Norway; 2 Stavanger University Hospital, Norway; 3 University of Bergen, Norway; 4 Norwegian Institute of Public Health, Bergen, Norway; 5 Aarhus University, Denmark

**Keywords:** ADHD, conduct problems, substance-related problems, adolescence, child and adolescent mental health services

## Abstract

**Background:**

Attention-deficit/hyperactivity disorder (ADHD) is a known risk factor for substance-related problems (SRP) during adolescence, but the nature of this relationship and the importance of co-occurring conduct problems are not fully understood.

**Methods:**

Data stem from a linked dataset between a large population-based survey conducted in 2012 of Norwegian adolescents aged 16 to 19, and registry-based data from specialized child and adolescent mental health services (*n* = 9,411).

**Results:**

Adolescents with “ADHD + high conduct problems” had increased risk of SRP (odds ratios = 2.37–10.14). Adolescents with “ADHD only” had very similar risk of SRP as adolescents from the general population with low symptoms of conduct problems. Relative to boys, girls with “ADHD + high conduct problems” appeared to have somewhat higher risk for SRP.

**Conclusion:**

The present study suggests that the risk for SRP among adolescent with ADHD is largely driven by co-existing conduct problems.

## Introduction

Attention-deficit/hyperactivity disorder (ADHD) is a neurodevelopmental disorder characterized by pervasive and impairing symptoms of inattention, hyperactivity and impulsivity (American Psychiatric Association, 2013). Among school-age children, the prevalence of this diagnosis is estimated to be between 3% and 9% (Esser et al., 1990; Scahill & Schwab-Stone, 2000; Szatmari et al., 1989). The disorder is associated with increased risk of school-related problems, psychosocial problems, and future unemployment (Agnew-Blais et al., 2018; Biederman et al., 1996). Large population-based studies have estimated that between 44% and 90% of children and adolescents with ADHD will have at least one comorbid disorder (Barkley, 1998; Biederman et al., 1991; Mitchison & Njardvik, 2019; Szatmari et al., 1989; Willcutt et al., 1999), as well as an increased risk of substance-related problems (SRP) (Agnew-Blais et al., 2018; Erskine et al., 2016; Franke et al., 2018; Heradstveit et al., 2018; Lee et al., 2011; Molina & Pelham, 2003; Yewers et al., 2005). Several studies have shown that disruptive behavior diagnoses—such as conduct disorders (CD) and oppositional-defiant disorders (ODD)—commonly co-exist with ADHD in children and adolescents (Elia et al., 2008; Pfiffner et al., 2005). These diagnoses imply problems related to difficulty to accept rules; aggressive behavior; and antisocial behaviors (Azeredo et al., 2018). Conduct problems is a relatively inclusive term that is commonly used to describe the presence of symptoms of a disruptive behavior disorder. A range of population-based studies of adolescents have linked conduct problems to SRP (Bukstein, 2000; Heradstveit et al., 2019; Pedersen et al., 2018; Tarter et al., 2006). Several reviews and meta-analyses have suggested that the increased risk of SRPs among adolescents with ADHD is largely explained by the presence of comorbid conduct problems (Flory & Lynam, 2003; Lee et al., 2011; Serra-Pinheiro et al., 2013). Few of these studies have, however, used data from specialized child and mental health services (CAMHS).

A recent Norwegian study reported that only 4% of adolescents in CAMHS had received a disruptive behavior diagnosis (Heradstveit et al., 2019). This rather low diagnostic rate in a clinical adolescent setting contrasts lifetime prevalence rates of disruptive disorders in the general population, which are estimated to be 12% among boys and 7% among girls with a median age-of-onset of 12 years (Nock et al., 2006). On the other hand, a population-based study corroborated that the estimated prevalence of a disruptive behavior disorder is fairly low in Norway, at around 2.5% in 8 to 10 years olds (Heiervang et al., 2007) and 1.3% in 10 to 14 years olds (Bøe et al., 2021). We have reasons to believe that not all individuals with conduct problems receive a formal disruptive behavior diagnosis, even if they are referred to CAMHS, and the use of self-reported measures of conduct problems may add valuable information of the distribution of potentially undetected disruptive behavior disorders in clinical settings. In the present study we will therefore investigate if self-reported conduct problems are more prevalent than indicated by the diagnostic prevalence provided by CAMHS, and if the severity of this behavior is associated with an increased risk for SRP among adolescents with an ADHD diagnosis.

Little is known regarding the influence of sex and negative life events on associations between ADHD, conduct problems, and SRP. Girls with disruptive behavior disorders tend to experience more social problems than boys (Carlson et al., 1997; Sihvola et al., 2011), and research on sex differences in outcomes related to ADHD and comorbid disruptive behavior disorders are called for (Gaub & Carlson, 1997). While several studies suggest that ADHD is a stronger risk factor for SRP for girls than boys (Norén Selinus et al., 2016; Sihvola et al., 2011), very few studies have investigated the possibility that co-existing conduct problems may affect the sexes differentially with respect to SRP. However, one study reported that ADHD symptoms were associated with SRP after accounting for conduct problems, notably only among girls (Sihvola et al., 2011). Negative life events are strongly associated with SRP, including exposure to interpersonal violence (Keyes et al., 2011), accidents and catastrophic events (Cerdá et al., 2011), and death among parents, siblings, or friends (Melhem et al., 2008), and the risk appears to increase along with the cumulative load of these stressful events (Anda et al., 2006; Ford et al., 2010). Negative life events has, despite the strong biological roots of ADHD (Goldstein et al., 2007), also been found to contribute significantly to the severity of both ADHD and conduct problems (Rydell, 2010). It may therefore be particularly useful to evaluate to what extent negative life events account for the association between ADHD, conduct problems, and SRP.

### The Present Study

The primary purpose of this paper was to assess if severity of self-reported conduct problems is associated with differential risk for SRP among adolescents diagnosed with ADHD in a Norwegian CAMHS-setting, and whether these associations differ across sexes. Specifically, we hypothesized that individuals with ADHD and high levels of conduct problems, used as a proxy for a CD, would have a significantly increased risk of SRP. Furthermore, we hypothesized that associations between ADHD and co-existing conduct problems and SRP would be stronger among girls than boys. Finally, we hypothesized that adjusting for negative life events would attenuate, but not fully account for, these associations.

## Methods

### Participants and Procedure

This study used data from a linkage between a large population-based study (the youth@hordaland [y@h]-survey) and registry-based data from CAMHS (the Norwegian Patient Registry [NPR]). This linkage has been described in previous studies (Heradstveit et al., 2019; Hysing et al., 2020).

The *y@h-survey* was conducted in 2012 and included adolescents aged 16 to 19 years living in Hordaland County in Western Norway. Ten thousand, two hundred fifty-seven adolescents participated in the survey, comprising 53% of the total adolescent population (*n* = 19,430). Prior to inclusion in the study, informed consent was retrieved from all participants and one school hour was used to complete the web-based questionnaire. Adolescents not going to school received the questionnaire by mail at their home address and child welfare service institutions and inpatient psychiatric hospitals were also contacted to let adolescents from these settings participate. Eight hundred and forty six adolescents that participated in the y@h (8.2% of the total number of respondents) did not consent to linkage with official registries. Therefore, 9,411 adolescents from the y@h-survey were available for the linkage with CAMHS data.

The *NPR* is the official registry on CAMHS use in Norway and includes information on psychiatric diagnoses, and data on the treatment provided for each patient. The majority of contacts with CAMHS in Norway comprise outpatient clinical consultations, including direct contact (i.e., face to face conversations between professional health worker(s), the adolescent, and/or the family) and indirect contact (i.e., co-operation between the professional health worker and the adolescent’s network, such as school personnel) (Indergård et al., 2019). Of the 9,411 eligible adolescents, 970 adolescents (10.3%) had received treatment with CAMHS according to the registry. For consenting participants, all register based CAMHS contact was extracted, and thus included information prior to, during, and following the time of survey participation. Data from CAMHS spanned from January 2008 to March 2018.

The Regional Committee for Medical and Health Research Ethics in Western Norway (2011/811/REK Vest; 2012/1467/REK Vest) and Norwegian Centre for Research Data (NSD; 371974 and 259631) approved the study. All management of data in the present study adhered to the General Data Protection Regulation (GDPR) directive and a Data Protection Impact Assessment was conducted for the linkage.

### Representativeness of the CAMHS-Sample

Approximately 5% of Norwegian adolescents below 18 years of age receive treatment in CAMHS yearly (Indergård et al., 2019). Data from a previous study using the linkage between the y@h-survey and CAMHS data from the NPR found that 9.1% of the individuals participating in the y@h had received treatment from CAMHS during the past 4 years (Heradstveit et al., 2019). With few exceptions, individuals who consented to registry-linkage with CAMHS were for the most part similar to the adolescents that did not consent (Heradstveit et al., 2019). Adolescents who refused consent had, however, somewhat higher alcohol consumption and self-reported symptoms of conduct problems (effect sizes = 0.11) and were slightly older (17.6 vs. 17.4 years).

### Materials

#### ADHD diagnosis and co-existing conduct problems

Clinical professionals assessed psychiatric diagnoses for each of the adolescents that received treatment from CAMHS. These diagnoses were coded on Axis 1 according to the ICD-10 diagnostic manual (WHO, 1992) and registered in CAMHS. Hyperkinetic disorders (i.e., diagnoses within the F90-chapter) were recorded for 177 individuals (18% of the individuals receiving treatment in CAMHS). These diagnoses were used as a proxy for an ADHD-diagnosis.

Self-reported conduct problems were measured in the y@h-survey using the self-reported 8-item scale for conduct disorders (CD) from the Diagnostic Interview Schedule for Children Predictive Scales (DPS) instrument (Lucas et al., 2001). The DPS-CD scale has been shown to define adolescents who are at high probability of meeting the diagnostic criteria for CD, using a cut-off score of 2 or more (Lucas et al., 2001). The Cronbach’s alpha coefficient for internal consistency of the DPS-CD scale in our sample was .74.

We created a variable for ADHD groups, in which individuals who received an ADHD diagnosis during their contact with CAMHS were categorized into the following three groups: (i) “ADHD only”; (ii) “ADHD + low conduct problems”; and (iii) “ADHD + high conduct problems.” The first group was defined as those who had no indications of conduct problems (score = 0 on the DPS-CD scale). The second group was defined as those who had a score of 1 on the DPS-CD scale. The third group was defined as those who had a score of 2 or more on the DPS-CD scale, as this cut-off is suggested to indicate a potential CD (Lucas et al., 2001). Seven individuals (4.0%) had missing responses on the scale and were excluded. Thus, this variable included 170 individuals with ADHD and distributed as follows: “ADHD only”: *n* = 89; “ADHD + low conduct problems”: *n* = 32; and “ADHD + high conduct problems”: *n* = 49. Individuals from the general population comprised the survey sample. This full survey sample consisted of 8,441 individuals, but after the exclusion of those without valid scores on the DPS-CD-scale (*n* = 170) and those with scores ≥1 on the DPS-CD scale (*n* = 2,141), the survey sample consisted of 6,130 individuals (72.6% of total).

#### Substance-related problems (SRP)

SRP were measured as part of the y@h-survey, with five variables that are thoroughly described in a previous article (Heradstveit et al., 2019). (1) *Illicit drug use* was based on a single item: “Have you ever tried hash, marijuana or other narcotic substances?” (Yes/No). (2) *High-level alcohol consumption* was based on a variable that added up five items measuring how many glasses of (i) beer, (ii) cider, (iii) wine, (iv) spirits, and (v) illegally distilled spirits the adolescents usually consumed during a period of 14 days. The variable separated those above the 80th sex-specific percentile on alcohol consumption among adolescents with any usual alcohol consumption, from those below. (3) *Frequent alcohol intoxication* was based on a single item: “Have you ever consumed so much alcohol that you were clearly intoxicated (drunk)?” The original item had five categories ranging from “No, never” to “Yes, more than 10 times.” Frequent alcohol intoxication was defined as drinking so much that one was clearly intoxicated more than 10 times, and on this basis, a dichotomous variable was created. (4) *Positive CRAFFT score* was based on the 6-item, validated CRAFFT scale (Knight et al., 2002). CRAFFT stands for the key words of the six items included in the scale—Car, Relax, Alone, Forget, Friends, Trouble. This scale has been designed to identify potential alcohol-and drug related problems among adolescents and has been demonstrated to have acceptable sensitivity and specificity at a cut-off value of ≥2 (Dhalla et al., 2011; Knight et al., 2002; Skogen et al., 2013). A dichotomous variable separating those above and below this CRAFFT cut-off value (≥2) was calculated. (5) Finally, an ordinal variable for level of *total symptoms of SRP* was constructed (ranging from 0 to 4), in which we summed up the number of positive scores on lifetime illicit drug use, high-level alcohol consumption, frequent alcohol intoxication, and positive CRAFFT-score.

##### Negative life events

*Negative life events* (NLE) were measured as part of the y@h-survey with eight items covering different events (Askeland et al., 2020). Four of the items were dichotomous; (1) “death of a parent/guardian,” (2) “death of a sibling,” (3) “death of a close friend,” and (4) “death of girlfriend/boyfriend.” Four items were ordinal: (5) Having encountered “a catastrophe or serious accident” had the following response categories: “No, never,” “yes, once,” to “Yes, more than once,” while (6) having experienced “violence from a grown up,” (7) having “witnessed someone you care about being exposed to violence from a grown up,” and (8) having experienced “unwanted sexual actions” had the following response categories: “No, never,” “Yes, once,” “Yes, a few times,” to “Yes, many times.” All the ordinal variables were dichotomized in order to differentiate between those answering “No, never” from those with positive responses. An ordinal NLE-variable (spanning from 0 to 8) was constructed by summing the eight dichotomous NLE-variables mentioned above.

#### Other variables

##### Disruptive behavior disorder-diagnoses

Disruptive behavior disorder diagnoses were retrieved from CAHMS and included all diagnoses from chapter F91 (“Conduct disorders”) in ICD-10. Hence, we summarized both CD diagnoses (F91.0–F91.2) and other disruptive behavior diagnoses, including ODD (F91.3). To secure that no F91-diagnoses were ignored, we also included F92 (“Mixed disorder of conduct and emotions,” which formally requires the presence of an F91.-diagnosis).

##### Duration of CAMHS-contact

Contact with CAMHS was defined as having a valid registration in the NPR. A continuous variable was constructed for the duration of treatment in CAMHS, which counted the number of months with a registered contact with the services. This variable spanned from 1 to 65 months (*M* = 11.45; *SD* = 10.33; median = 8; interquartile range = 4–16).

##### Sociodemographic variables

Sex and age of all participants were retrieved from the personal identification number from the Norwegian Population Registry and were available for all participants of the y@h-sample. Three measures of self-reported socioeconomic status were used and included the perceived economic well-being of the family (response categories: “poorer than others,” “equal to others,” and “better than others”), and maternal and paternal educational attainment (categorized into: “primary school,” “high school,” and “college/university”). Three variables were also included that separated those with Norway as their country of origin from those born outside of Norway. These variables related to (1) the adolescent’s country of origin; (2) the adolescents mother’s country of origin; and (3) the adolescent’s father’s country of origin defined as Norway or “other country.” One variable specified whether the adolescents’ parents were living together.

### Statistical Analyses

First, we provided descriptive statistics for the sociodemographic variables and self-reported conduct problems. Two separate analytical approaches were used in this respect. In approach 1, all variables were compared between the non-clinical y@h-population (i.e., the survey sample) and adolescents with ADHD (i.e., the clinical sample). In approach 2, we described the same variables across the three ADHD-subgroups using the “ADHD only” group as reference level against “ADHD + low conduct problems,” and “ADHD + high conduct problems, respectively. These analyses were conducted stepwise, comparing “ADHD only” with each of the other ADHD-subgroups separately. The analyses are summarized in Table 1. We also analyzed rates of negative life events (Table 2) and duration of treatment in CAMHS across the three ADHD-subgroups (not shown). Independent samples *t*-tests were used to compare means between the groups in all these analyses, while Pearson chi-square tests were used to compare differences across categorical variables. Second, we described comorbidity with formal disruptive behavior disorder diagnoses across the subgroups of ADHD. Third, we calculated the unadjusted rates of total symptoms of SRP with 95% confidence interval in the three ADHD-subgroups as well as in the survey sample and visualized these rates in a figure. Finally, we used logistic and ordered logistic regression analyses to investigate associations between ADHD and SRP stratified by each of the three ADHD-groups with the survey sample as base level (Table 3). In Model 1, associations were unadjusted. In Model 2, we included the adjustment for sex and age. In Model 3, we also included negative life events. In all cases where we found a positive association in Model 1, we calculated how much the inclusion of co-variates reduced the strength of this association by dividing the log odds from Model 3 with the log odds from Model 1. Using the same method, we also compared Model 3 with Model 2 to calculate how much the inclusion of negative life events reduced the strength of the association. All data were analyzed using STATA version 15 (StataCorp, 2017).

**Table 1.** Descriptive Characteristics of the Sample (*n* = 9,411).

	Study sample^1,2^		Subgroups of ADHD (*n* = 170)^3,4^		
	Survey sample (*n* = 6,130)^5^	CAMHS: ADHD-diagnoses (*n* = 170)	ADHD only (*n* = 89)	ADHD + low conduct problems (*n* = 32)	ADHD + high conduct problems (*n* = 49)
Sex: Boys, % (*n*)	44.7 (3,362)	54.1 (92)*	46.1 (41)	59.4 (19)	65.3 (32)*
Age, mean (SD)	17.45 (0.84)	17.28 (0.82)*	17.36 (0.84)	17.30 (0.77)	17.13 (0.81)
Perceived family economic well-being		*			
Poorer than others, % (*n*)	5.8 (347)	**10.2 (17)**	6.8 (6)	20.0 (6)	10.2 (5)
Equal to others, % (*n*)	69.3 (4,173)	**61.7 (103)**	62.5 (55)	63.3 (19)	59.2 (29)
Better than others, % (*n*)	24.9 (1,499)	**28.1 (47)**	30.7 (27)	16.7 (5)	30.6 (15)
Mothers educational level		**			
Basic, % (*n*)	9.1 (431)	**18.0 (21)**	14.5 (9)	25.0 (5)	20.0 (7)
Intermediate, % (*n*)	41.0 (1,943)	**41.0 (48)**	40.3 (25)	40.0 (8)	42.9 (15)
High, % (*n*)	49.9 (2,362)	**41.0 (48)**	45.2 (28)	35.0 (7)	37.1 (13)
Fathers educational level		**			
Basic, % (*n*)	9.5 (442)	**18.8 (21)**	16.1 (10)	25.0 (5)	20.0 (6)
Intermediate, % (*n*)	46.5 (2,161)	**50.9 (57)**	51.6 (32)	45.0 (9)	53.3 (16)
High, % (*n*)	44.0 (2,043)	**30.4 (34)**	32.3 (20)	30.0 (6)	26.7 (8)
Country of origin: Norway					
Self, % (*n*)	94.6 (5,741)	94.0 (157)	93.1 (81)	96.9 (31)	93.8 (45)
Mother, % (*n*)	91.2 (5,589)	97.7 (166)**	96.6 (86)	96.9 (31)	100.0 (49)
Father, % (*n*)	90.2 (5,512)	91.7 (154)	92.1 (82)	87.1 (27)	93.8 (45)
Parents living together, % (*n*)	71.1 (4,181)	44.7 (71)***	45.2 (38)	56.3 (18)	34.9 (15)

*Note.* Bold fonts denote statistically significant group differences between given group and reference group at *p*-values: *p < .05. **p < .01. ***p < .001. CAMHS = child and adolescent mental health services; ADHD = attention-deficit/hyperactivity disorder.

1 Reference: survey sample (i.e., non-clinical sample of the youth@hordaland-survey).

2 Seven hundred and ninety three adolescents received CAMHS, but without having ADHD-diagnoses, and are not shown in this table.

3 Reference: “ADHD only” (*n* = 89).

4 Seven adolescents had missing responses on the DPS-CD scale and were not assigned to any subgroup of ADHD.

5 Excluding 2,311 individuals, either due to missing score on the DPS-CD scale (*n* = 170) or a DPS-CD-score at ≥1 (*n* = 2,141).

**Table 2.** Rates of Negative Life Events Across the Three Defined ADHD Subgroups (*n* = 170).

	ADHD only (*n* = 89) % (*n*)	ADHD + low conduct problems (*n* = 32) % (*n*)	ADHD + high conduct problems (*n* = 49) % (*n*)
Death of someone close (any)	27.7 (18)	24.0 (6)	33.3 (11)
Catastrophe or serious accident	18.8 (16)	<5	22.7 (10)
Interpersonal violence (any)	31.0 (26)	48.4 (15)	59.1 (26)**
Experienced violence from grown up	16.7 (14)	25.8 (8)	43.2 (19)**
Witnessed violence from grown up	23.8 (20)	29.0 (9)	45.5 (20)*
Unwanted sexual actions	15.3 (13)	19.4 (6)	13.6 (6)
Total, mean (*SD*)	0.92 (1.20)	1.03 (1.09)	1.39 (1.47)*
Total, median (interquartile range)	0 (0, 2)	1 (0, 2)**	1 (0, 3)**

*Note.* Reference group: ADHD only. Bold fonts denote statistically significant group differences between given group and reference group at *p*-values: *p < .05. **p < .01. Rates are not calculated for cells with less than *n* = 5.

**Table 3.** Associations Between the Defined ADHD Subgroups Stratified by Conduct Problems and SRP (*n* = 6,300).

	Model 1			Model 2			Model 3		
	Unadjusted			Adjusted for age and sex			Adjusted for age, sex, and negative life events		
	ADHD only OR (95% CI)	ADHD + low conduct problems OR (95% CI)	ADHD + high conduct problems OR (95% CI)	ADHD only AOR (95% CI)	ADHD + low conduct problems AOR (95% CI)	ADHD + high conduct problems AOR (95% CI)	ADHD only AOR (95% CI)	ADHD + low conduct problems AOR (95% CI)	ADHD + high conduct problems AOR (95% CI)
Tried illicit drugs	1.40 (0.64, 3.05)	2.34 (0.81, 6.71)	10.14 (5.56, 18.51)***	1.47 (0.67, 3.24)	2.52 (0.86, 7.35)	11.35 (5.95, 21.66)***	1.09 (0.49, 2.46)	1.90 (0.64, 5.68)	7.47 (3.78, 14.77)***
High-level alcohol consumption	0.38 (0.12, 1.21)	1.39 (0.49, 4.00)	2.49 (1.19, 5.22)*	0.39 (0.12, 1.26)	1.57 (0.54, 4.54)	3.39 (1.59, 7.24)**	0.31 (0.10, 1.01)	1.28 (0.44, 3.75)	2.49 (1.14, 5.44)*
Frequent alcohol intoxication	1.36 (0.81, 2.30)	1.51 (0.65, 3.49)	2.37 (1.29, 4.37)**	1.53 (0.88, 2.65)	1.86 (0.77, 4.47)	3.00 (1.52, 5.93)**	1.30 (0.74, 2.28)	1.56 (0.64, 3.79)	2.27 (1.12, 4.58)*
Positive CRAFFT score	0.82 (0.43, 1.55)	2.31 (1.06, 5.04)*	4.52 (2.50, 8.18)***	0.84 (0.44, 1.60)	2.66 (1.21, 5.88)*	5.38 (2.88, 10.05)***	0.62 (0.32, 1.21)	2.01 (0.89, 4.54)	3.52 (1.81, 6.84)***
Total symptoms of SRP	1.17 (0.72, 1.88)	1.75 (0.86, 3.56)	6.00 (3.44, 10.48)***	1.09 (0.67, 1.78)	1.93 (0.96, 3.89)	7.46 (4.15, 13.44)***	0.85 (0.52, 1.40)	1.50 (0.74, 3.06)	5.12 (2.80, 9.36)***

*Note.* Reference: survey sample (*n* = 6,130). Bold fonts denote statistically significant associations at *p*-values: *p < .01, **p < .001. SRP = substance-related problems. CRAFFT = acronym for the six items that comprise this screening scale for alcohol/drug-related problems among adolescents. A score of ≥2 on this scale is considered a positive score.

## Results

### Descriptive Characteristics of the Sample

As shown in Table 1, adolescents diagnosed with ADHD comprised more boys and were slightly younger than adolescents in the independent survey sample. They had lower perceived family economic well-being and had parents with lower educational levels, more often mothers born in Norway (98% vs. 91%), and less often parents living together (45% vs. 69%). Individuals across the subgroups of ADHD were for the most part similar in terms of sociodemographic characteristic and they received treatment of similar duration (mean duration across groups ranging from 15.9 to 16.7 months; median duration ranging from 14.5 to 16; not shown). However, adolescents with “ADHD + high conduct problems” were more often boys (*p* < .05) than in the “ADHD only” group.

Of the 170 adolescents with an ADHD diagnosis, 8% (*n* = 13) had a comorbid disruptive behavior disorder diagnosis according to CAMHS. In contrast, 29% (*n* = 49) of the adolescents with ADHD were screen positive for CD (i.e., they scored ≥2 on the DPS-CD questionnaire), compared with 10% in the full survey sample (*n* = 8,441). Rates of diagnosed disruptive behavior disorders were low across all subtypes of ADHD, with 10% in the “ADHD + high conduct problems” group, and <5% in the “ADHD + low conduct problems,” and the “ADHD only” group.

The “ADHD + high conduct problems” group had a higher mean number of negative life events compared with the “ADHD only” group (*M* = 1.39 vs. 0.92; Table 2). Specifically, adolescents in the “ADHD + high conduct problems” reported more often to have experienced interpersonal violence (59% vs. 31%). There were no significant differences in negative life events between the “ADHD only” and “ADHD + low conduct problems” group.

### Substance-Related Problems Across Subgroups of ADHD

**Figure 1.**
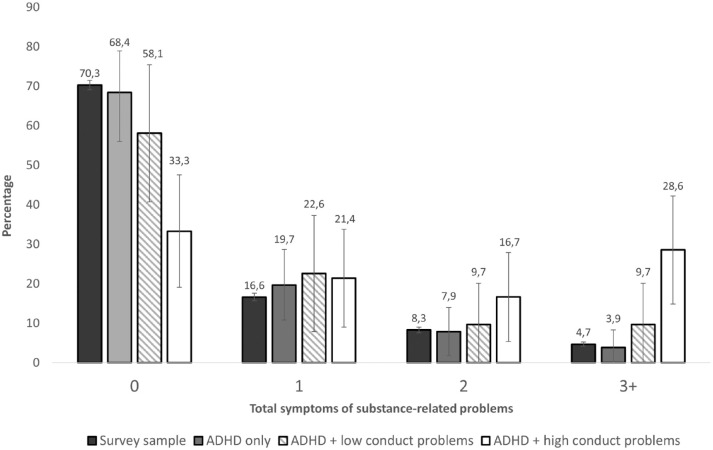
Unadjusted rates of total symptoms of substance-related problems in adolescents with ADHD-diagnoses, stratified by co-existing conduct problems, compared with the survey sample (*n* = 9,441).

Rates of total symptoms of SRP differed considerably across the ADHD subgroups (Figure 1 and Supplemental Table S1). Compared to the survey sample, the “ADHD only” group had very similar rates of SRP while the “ADHD + low conduct problems” group had slightly higher SRP rates. The “ADHD + high conduct problems” group had considerably higher rates of SRP than both the survey sample and the “ADHD only” group. Specifically, 28.6% (95% CI [14.9, 42.2]) of the adolescents with “ADHD + high conduct problems” had three or more indicators of SRP, compared to 4.7% (95% CI [4.2, 5.3]) in the survey sample and 3.9% (95% CI [0.0, 8.3]) in the “ADHD only”-group.

As outlined in Table 3, the “ADHD + high conduct problems” group had significantly heightened odds of SRP across all measures (odds ratios [ORs] ranging from 2.37 to 10.14). We found no heightened risk for SRP in the other subgroups of ADHD, except for higher odds for a positive CRAFFT score in the “ADHD + low conduct problems” group in Model 1 (OR = 2.31) and Model 2 (OR = 2.66). The full inclusion of co-variates (i.e., sex, age, and negative life events) attenuated the associations with SRP for the “ADHD + high conduct problems” group. Specifically, for the “ADHD + high conduct problems” group, the full inclusion of co-variates reduced the strength of the odds ratios with 13% for illicit drug use; 0% for high-level alcohol consumption; 5% for frequent alcohol intoxication; 17% for a positive CRAFFT score; and 9% for total symptoms of SRP compared to the unadjusted model/Model 1. On the other hand, the specific inclusion of the covariate for negative life events (i.e., comparing the change in the estimates from Model 2 to Model 3) reduced the strength of the odds ratios with 17% to 25% across the different measures of SRP. For the “ADHD + low conduct problems” group, the full inclusion of co-variates reduced the strength of the odds ratio for a positive CRAFFT score with 17% from Model 1 to 3, and with 29% from Model 2 to 3.

The low sample size of the ADHD-subgroups prevented us from conducting formal interaction analyses between the ADHD-subgroups and sex on the associations with SRP. However, a visual inspection of our data revealed that rates of total symptoms of SRP were heightened among both boys and girls with “ADHD + high conduct problems,” but most pronounced among girls. Specifically, 21.4% (95% CI [6.2, 36.6]) of boys with “ADHD + high conduct problems” had three or more indicators of total symptoms of SRP compared with 4.8% (95% CI [4.0, 5.6]) in the non-clinical male survey sample and 5.9% (95% CI [0.0, 13.8]) of those with ADHD only, while 42.9% (95% CI [16.9, 68.8]) of girls with “ADHD + high conduct problems” had three or more indicators of total symptoms of SRP compared with 4.6% (95% CI [3.9, 5.4]) in the non-clinical female survey sample and 2.4% (95% CI [0.0, 7.0]) of those with ADHD only. See Supplemental Table S1 for details.

## Discussion

In the present study, we aimed to investigate SRP among subgroups of adolescents with ADHD characterized by different severity levels of conduct problems, using unique data with a linkage between a large population-based survey of Norwegian adolescents and an official registry on CAMHS-use.

The main finding of the present study was a considerably increased risk of SRP among adolescents with an ADHD diagnosis in combination with a high self-reported conduct problems score. This finding was evident for all the included measures of SRP, although with varying magnitude. For example, adolescents with ADHD and high conduct problems had seven-fold increased odds for illicit drug use compared with the survey sample, even after adjustment for negative life events. On the other hand, adolescents with “ADHD only” had similar risk compared with the expected SPR rate in the survey sample. This pattern was evident across all the included measures of SRP. Our findings thus lend support to the notion that the risk of SRP among ADHD-diagnosed adolescents can largely be attributed to co-existing conduct problems (Disney et al., 1999; Lee et al., 2011; Serra-Pinheiro et al., 2013), and that ADHD in itself does not increase the risk of adolescent illicit drug use beyond the effect of conduct-related disorders (Serra-Pinheiro et al., 2013).

A range of previous studies have reported that adolescents with ADHD have a high probability of co-existing conduct problems (Barkley, 1998; Biederman et al., 1991; Mitchison & Njardvik, 2019; Szatmari et al., 1989; Willcutt et al., 1999). This was strongly supported by results in the current study. Self-reported conduct problems were nearly three times more common among adolescents with ADHD compared to estimates in the general population. On the other hand, whereas 29% of all adolescents with ADHD were screen positive for CD according to the DPS-CD questionnaire, only 10% of these screen-positive adolescents had a formally diagnosed disruptive behavior disorder. This finding may be surprising considering population-based studies that generally report comorbidity rates between ADHD and disruptive behavior problems in the range from 20% to 56% (Barkley, 1998; Biederman et al., 1991). It is likely that this discrepancy may reflect a degree of under-detection of disruptive behavior disorders in CAMHS or clinicians being hesitant to categorize the adolescents with such a disorder.

Negative life events were reported more often by adolescents with an ADHD diagnosis in combination with conduct problems compared with those without these co-existing problems. However, negative life events only modestly attenuated the risk for SRP among adolescents with ADHD and high conduct problems. Thus, our findings suggest that the risk for SRP among adolescents with combined ADHD and conduct problems may not be restricted to those exposed to higher levels of negative life events. More specifically, following the inclusion of negative life events, all associations with SRP were still significant, and the strength of the odds ratios were reduced with 17% to 25%. Therefore, negative life events did not appear to explain the increased risk of SRP among adolescents with combined ADHD and conduct problems, but it should nevertheless be regarded as a relevant factor in the association between ADHD and SRP. Furthermore, the high prevalence of negative life events among adolescents with ADHD was most pronounced in relation to interpersonal violence. Potential explanations for this finding include: (1) that negative life events are part of the etiological mechanisms for development of conduct problems in adolescents with ADHD; (2) that a lifestyle characterized by disruptive behaviors increase the risk of exposure to certain negative life events; or (3) that unmeasured third variables account for this finding. While the present study did not investigate mechanisms that may explain the link between negative life events and conduct problems, our findings lend some support to a CAMHS-study which reported that exposure to interpersonal violence—but not non-interpersonal traumas—was related to more severe disruptive behavioral problems, independent of demographics and psychiatric diagnoses (Ford et al., 2011).

The presence of ADHD and co-existing conduct problems was associated with SRP for both boys and girls. Although we did not have sufficient subgroup sample sizes for formal investigations of sex differences, our findings suggest that the association between “ADHD + high conduct problems” and SRP may be somewhat stronger among girls. A previous study of co-occurring ADHD and disruptive disorders reported that despite similar or less behavioral dysfunction, girls with disruptive disorders experienced more social problems than boys (Carlson et al., 1997). A population-based study of adolescent twins reported that although no significant sex differences were found in the effects of ADHD and conduct disorder on SRP, girls with ADHD still seem to be at slightly higher risk for SRP than boys with ADHD (Disney et al., 1999). We recommend that future studies further elaborate on potential sex differences in risk for SRP among adolescents with ADHD with and without co-existing conduct problems. Of note, there was a relatively equal sex distribution among adolescents with an ADHD diagnosis in the present study, with only a slightly higher rate in boys than girls—translating to a sex ratio of 1.2:1. In comparison, several previous population-based studies have estimated the boys/girls ADHD ratio to be between 2:1 and 3:1 in community samples and from 2:1 to 9:1 in clinical samples (Arcia & Conners, 1998; Bruchmüller et al., 2012; Huss et al., 2008). A possible explanation of this modest sex ratio in our study is that many boys in our material could potentially have received an ADHD-diagnosis at a contact with CAMHS prior to the age of 12, which was the minimum age of registered contact. Official registry data from Norwegian CAMHS seem to support this interpretation, as far more primary school-aged boys than girls are referred to CAMHS with ADHD as the diagnosis of suspicion (Indergård et al., 2019). On the other hand, boys were overrepresented among the individuals with ADHD and severe self-reported conduct problems, with a sex-ratio at approximately 2:1.

Finally, the present study highlights several sociodemographic characteristics of the sample that should be considered. Previous investigations have reported that ADHD prevalence rates are higher among adolescents from low SES families, in terms of both family income and parental education (Huss et al., 2008). This pattern was also observed in the present study, as adolescents with an ADHD diagnosis had a SES profile characterized by both a worse family economy and lower parental education compared with the survey sample. Adolescents with an ADHD diagnosis also more often had native-born mothers compared with the survey sample. This finding may either suggest a representativeness issue of the present sample, and/or that mothers with a history of migration has a lower health service utilization rate on behalf of offspring with ADHD symptoms. A study by Huss et al. (2008) found that families with a history of migration reported fewer ADHD-diagnoses for their children, but also more severe ADHD-symptoms. The authors suggested several possible explanations, which also may apply to the findings of the present study, including migrant-specific patterns of health service utilization, migrant-specific low rates of the ADHD-diagnosis, or cultural differences in the tolerance of symptoms.

### Implications for Clinical Practice and Research

Few adolescents with ADHD had received a comorbid disruptive behavior disorder diagnosis, even among those reporting symptoms indicating CD in the “ADHD + high conduct problems” group. The high levels of self-reported conduct problems in this group, along with the increased risk for SRP, suggest that under-detection in CAMHS may leave adolescents at high-risk for unidentified SRP. Behavior problems associated with disruptive disorders are shown to be a potent risk factor for SRP, and particularly illicit drug use (Bukstein, 2000; Heradstveit et al., 2019; Pedersen et al., 2018; Tarter et al., 2006). On the other hand, the low prevalence of diagnosed disruptive behavior disorders in the ADHD-group may also indicate a reluctance toward giving this diagnosis to youth, due to the potentially stigmatizing nature of this disorder. The results underline the need for CAMHS and other relevant health services to enhance identification of adolescents with ADHD and severe conduct problems, and by this ensure access to interventions that may contribute to break negative cycles related to substance abuse (Hogue et al., 2014).

### Strengths and Limitations

A strength of the present study was the linkage between a large population-based sample and official registry data from CAMHS in which diagnoses were set by health professionals after thorough examinations. The use of a validated scale for conduct problems to assess self-reported symptoms at different severity levels and the inclusion of relevant co-variates, provide further strengths to the study.

The study also had some limitations. The use of hyperkinetic disorder diagnosis to define ADHD prevent more detailed studies of the inattentive and hyperactive/impulsive presentations described in the DSM-5 (American Psychiatric Association, 2013). The relatively low size of the subgroup samples yields a relatively low precision in the point estimates. With small samples and weak statistical power, results are generally less reliable even when they are statistically significant (Button et al., 2013). However, in the present study, the analyses were founded on an empirically informed and a priori determined hypothesis of a higher risk of SRP in adolescents with combined ADHD and conduct problems, and the strong support of this hypothesis is thus a plausible finding. Nevertheless, we advise that the low sample size of the ADHD-subgroups should be considered when interpreting our findings. The generalizability of our findings to CAMHS-settings in other countries is also unknown. We therefore advise to interpret the relative lack of sociodemographic differences across the subgroups of ADHD with caution, and to validate our findings on associations between subgroups of ADHD and SRP with larger samples.

The response rate of the population-based y@h-study was 53%. Our CAMHS-sample was retrieved from a linkage between the y@h and an official registry and does not comprise all adolescents receiving psychiatric treatment in the target population. SES is higher among the adolescents participating in the y@h-survey than expected from national statistics of the general Norwegian population (Bøe et al., 2017). Therefore, we cannot rule out that our study sample was somewhat more healthy than the true population of adolescents in CAMHS, as SES is associated with prevalence of psychiatric disorders (Bøe et al., 2012). Also, adolescents who did not consent to the registry-linkage, and therefore were omitted from our sample, have slightly higher alcohol consumption and self-reported conduct problems compared with the included adolescents (Heradstveit et al., 2019). These limitations may potentially bias the prevalence rates in our study sample. However, representativeness issues are less prone to affect associations between variables (Wolke et al., 2009), and the strong association between “ADHD + high conduct problems” and SRP is highly unlikely to be a result of selective participation. Another limitation was that some individuals in the y@h-sample did not consent to the linkage with registry-data, which may limit the representativeness of our sample. As the adolescents who did not provide consent were fairly similar to the y@h-sample (Heradstveit, 2019), we do not expect that exclusion of participants due to non-consent have seriously biased our results.

Finally, a range of other factors beyond those included in the present study are likely to affect the association between ADHD and SRP, such as adverse psychosocial conditions, negative peer influence, dropout from school, and previous experience with substance use. To study these factors was, however, beyond the scope of the present study. Although the present study reported heightened levels of exposure to interpersonal violence in the “ADHD + high conduct problems” group, our study did not explore potential mechanisms behind this link. Thus, we recommend future studies to investigate the role of negative life events more thoroughly, particularly exposure to interpersonal violence, on functional outcomes in adolescents with ADHD and conduct problems at different severity levels.

## Conclusion

In the present register-linked population-based study, there was a high rate of self-reported conduct problems among adolescents with ADHD. Nearly one-third of the adolescents with an ADHD diagnosis reported conduct problems at a severity level indicating the presence of a conduct disorder, but still only a minority of these screen-positive adolescents (10%) received any registered disruptive behavior disorder diagnosis in CAMHS. These findings may point to a need for improved detection of comorbid conduct disorders in adolescents with ADHD-diagnoses in CAMHS. Adolescents with “ADHD + high conduct problems” had a considerably increased risk of SRP. This risk remained significant after the adjustment for sex, age, and negative life events. The present study thus supports the notion that the risk for SRP among adolescent with ADHD may be due to co-existing conduct problems (Disney et al., 1999; Lee et al., 2011; Serra-Pinheiro et al., 2013). Relative to boys, girls with “ADHD + high conduct problems” appeared to have somewhat higher risk for SRP.

## Supplemental Material

sj-docx-1-jad-10.1177_10870547221105063 – Supplemental material for Substance-Related Problems in Adolescents with ADHD-Diagnoses: The Importance of Self-Reported Conduct Problems Click here for additional data file. Supplemental material, sj-docx-1-jad-10.1177_10870547221105063 for Substance-Related Problems in Adolescents with ADHD-Diagnoses: The Importance of Self-Reported Conduct Problems by Ove Heradstveit, Kristin Gärtner Askeland, Tormod Bøe, Astri Johansen Lundervold, Irene Bircow Elgen, Jens Christoffer Skogen, Mads Uffe Pedersen and Mari Hysing in Journal of Attention Disorders

## References

[bibr1-10870547221105063] Agnew-Blais J. C. Polanczyk G. V. Danese A. Wertz J. Moffitt T. E. Arseneault L. (2018). Young adult mental health and functional outcomes among individuals with remitted, persistent and late-onset ADHD. The British Journal of Psychiatry, 213(3), 526–534. 29957167 10.1192/bjp.2018.97 PMC6098692

[bibr2-10870547221105063] American Psychiatric Association. (2013). Diagnostic and statistical manual of mental disorders: DSM-5. Washington, D.C.: American Psychiatric Association.

[bibr3-10870547221105063] Anda R. F. Felitti V. J. Bremner J. D. Walker J. D. Whitfield C. Perry B. D. Dube S. R. Giles W. H. (2006). The enduring effects of abuse and related adverse experiences in childhood. A convergence of evidence from neurobiology and epidemiology. European Archives of Psychiatry and Clinical Neuroscience, 256(3), 174–186. 16311898 10.1007/s00406-005-0624-4 PMC3232061

[bibr4-10870547221105063] Arcia E. Conners C. K. (1998). Gender differences in ADHD? Journal of Developmental & Behavioral Pediatrics, 19, 77–83. 9584935 10.1097/00004703-199804000-00003

[bibr5-10870547221105063] Askeland K. G. Bøe T. Breivik K. La Greca A. M. Sivertsen B. Hysing M. (2020). Life events and adolescent depressive symptoms: Protective factors associated with resilience. PLoS One, 15(6), e0234109. 32502163 10.1371/journal.pone.0234109 PMC7274383

[bibr6-10870547221105063] Azeredo A. Moreira D. Barbosa F. (2018). ADHD, CD, and ODD: Systematic review of genetic and environmental risk factors. Research in Developmental Disabilities, 82, 10–19. 29361339 10.1016/j.ridd.2017.12.010

[bibr7-10870547221105063] Barkley R. A. (1998). Attention-deficit hyperactivity disorder. Scientific American, 279(3), 66–71. 10.1038/scientificamerican0998-66 9725940

[bibr8-10870547221105063] Biederman J. Faraone S. V. Milberger S. Jetton J. G. Chen L. Mick E. Greene R. W. Russell R. L. (1996). Is childhood oppositional defiant disorder a precursor to adolescent conduct disorder? Findings from a four-year follow-up study of children with ADHD. Journal of the American Academy of Child and Adolescent Psychiatry, 35(9), 1193–1204. 8824063 10.1097/00004583-199609000-00017

[bibr9-10870547221105063] Biederman J. Newcorn J. Sprich S. (1991). Comorbidity of attention deficit hyperactivity disorder with conduct, depressive, anxiety, and other disorders. American Journal of Psychiatry, 148(5), 564–577. 2018156 10.1176/ajp.148.5.564

[bibr10-10870547221105063] Bruchmüller K. Margraf J. Schneider S. (2012). Is ADHD diagnosed in accord with diagnostic criteria? Overdiagnosis and influence of client gender on diagnosis. Journal of Consulting and Clinical Psychology, 80(1), 128–138. 22201328 10.1037/a0026582

[bibr11-10870547221105063] Bukstein O. G. (2000). Disruptive behavior disorders and substance use disorders in adolescents. Journal of Psychoactive Drugs, 32(1), 67–79. 10801069 10.1080/02791072.2000.10400213

[bibr12-10870547221105063] Button K. S. Ioannidis J. P. Mokrysz C. Nosek B. A. Flint J. Robinson E. S. Munafò M. R. (2013). Power failure: why small sample size undermines the reliability of neuroscience. Nature Reviews Neuroscience, 14(5), 365–376. 23571845 10.1038/nrn3475

[bibr13-10870547221105063] Bøe T. Heiervang E. R. Stormark K. M. Lundervold A. J. Hysing M. (2021). Prevalence of psychiatric disorders in Norwegian 10-14-year-olds: Results from a cross-sectional study. PLoS One, 16(3), e0248864. 33740026 10.1371/journal.pone.0248864 PMC7978367

[bibr14-10870547221105063] Bøe T. Skogen J. C. Sivertsen B. Hysing M. Petrie K. J. Dearing E. Zachrisson H. D. (2017). Economic volatility in childhood and subsequent adolescent mental health problems: A longitudinal population-based study of adolescents. BMJ Open, 7(9), e017030. 10.1136/bmjopen-2017-017030 PMC5623474 28928191

[bibr15-10870547221105063] Bøe T. Øverland S. Lundervold A. J. Hysing M. (2012). Socioeconomic status and children’s mental health: Results from the Bergen Child Study. Social Psychiatry and Psychiatric Epidemiology, 47(10), 1557–1566. 22183690 10.1007/s00127-011-0462-9

[bibr16-10870547221105063] Carlson C. L. Tamm L. Gaub M. (1997). Gender differences in children with ADHD, ODD, and co-occurring ADHD/ODD identified in a school population. Journal of the American Academy of Child and Adolescent Psychiatry, 36(12), 1706–1714. 9401332 10.1097/00004583-199712000-00019

[bibr17-10870547221105063] Cerdá M. Tracy M. Galea S. (2011). A prospective population based study of changes in alcohol use and binge drinking after a mass traumatic event. Drug and Alcohol Dependence, 115(1-2), 1–8. 10.1016/j.drugalcdep.2010.09.011 PMC3039709 20977977

[bibr18-10870547221105063] Dhalla S. Zumbo B. D. Poole G. (2011). A review of the psychometric properties of the CRAFFT instrument: 1999-2010. Current Drug Abuse Reviews, 4(1), 57–64. 21466499 10.2174/1874473711104010057

[bibr19-10870547221105063] Disney E. R. Elkins I. J. McGue M. Iacono W. G. (1999). Effects of ADHD, conduct disorder, and gender on substance use and abuse in adolescence. American Journal of Psychiatry, 156(10), 1515–1521. 10518160 10.1176/ajp.156.10.1515

[bibr20-10870547221105063] Elia J. Ambrosini P. Berrettini W. (2008). ADHD characteristics: I. Concurrent co-morbidity patterns in children & adolescents. Child and Adolescent Psychiatry and Mental Health, 2(1), 15–19. 18598351 10.1186/1753-2000-2-15 PMC2500004

[bibr21-10870547221105063] Erskine H. E. Norman R. E. Ferrari A. J. Chan G. C. K. Copeland W. E. Whiteford H. A. Scott J. G. (2016). Long-term outcomes of attention-deficit/hyperactivity disorder and conduct disorder: A systematic review and meta-analysis. Journal of the American Academy of Child and Adolescent Psychiatry, 55(10), 841–850. 27663939 10.1016/j.jaac.2016.06.016

[bibr22-10870547221105063] Esser G. Schmidt M. H. Woerner W. (1990). Epidemiology and course of psychiatric disorders in school-age children–Results of a longitudinal study. Journal of Child Psychology and Psychiatry, 31(2), 243–263. 2312652 10.1111/j.1469-7610.1990.tb01565.x

[bibr23-10870547221105063] Flory K. Lynam D. R. (2003). The relation between attention deficit hyperactivity disorder and substance abuse: What role does conduct disorder play? Clinical Child and Family Psychology Review, 6(1), 1–16. 12659448 10.1023/a:1022260221570

[bibr24-10870547221105063] Ford J. D. Elhai J. D. Connor D. F. Frueh B. C. (2010). Poly-victimization and risk of posttraumatic, depressive, and substance use disorders and involvement in delinquency in a national sample of adolescents. Journal of Adolescent Health, 46(6), 545–552. 10.1016/j.jadohealth.2009.11.212 20472211

[bibr25-10870547221105063] Ford J. D. Gagnon K. Connor D. F. Pearson G. (2011). History of interpersonal violence, abuse, and nonvictimization trauma and severity of psychiatric symptoms among children in outpatient psychiatric treatment. Journal of Interpersonal Violence, 26(16), 3316–3337. 21362676 10.1177/0886260510393009

[bibr26-10870547221105063] Franke B. Michelini G. Asherson P. Banaschewski T. Bilbow A. Buitelaar J. K. Cormand B. Faraone S. V. Ginsberg Y. Haavik J. Kuntsi J. Larsson H. Lesch K. P. Ramos-Quiroga J. A. Réthelyi J. M. Ribases M. Reif A. (2018). Live fast, die young? A review on the developmental trajectories of ADHD across the lifespan. European Neuropsychopharmacology, 28(10), 1059–1088. 30195575 10.1016/j.euroneuro.2018.08.001 PMC6379245

[bibr27-10870547221105063] Gaub M. Carlson C. L. (1997). Gender differences in ADHD: A meta-analysis and critical review. Journal of the American Academy of Child and Adolescent Psychiatry, 36(8), 1036–1045. 9256583 10.1097/00004583-199708000-00011

[bibr28-10870547221105063] Goldstein L. H. Harvey E. A. Friedman-Weieneth J. L. Pierce C. Tellert A. Sippel J. C. (2007). Examining subtypes of behavior problems among 3-year-old children, part II: Investigating differences in parent psychopathology, couple conflict, and other family stressors. Journal of Abnormal Child Psychology, 35(1), 111–123. 17226095 10.1007/s10802-006-9088-x

[bibr29-10870547221105063] Heiervang E. , et al. (2007). Psychiatric disorders in Norwegian 8-to 10-year-olds: An epidemiological survey of prevalence, risk factors, and service use. Journal of the American Academy of Child and Adolescent Psychiatry, 46(4), 438–447. 17420678 10.1097/chi.0b013e31803062bf

[bibr30-10870547221105063] Heradstveit O. , et al. (2018). Prospective associations between childhood externalising and internalising problems and adolescent alcohol and drug use: The Bergen Child Study. Nordic Studies on Alcohol and Drugs, 35(5), 357–371. 10.1177/1455072518789852 PMC7434147 32934538

[bibr31-10870547221105063] Heradstveit O. (2019). Alcohol- and drug use among adolescents. School-related problems, childhood mental health problems, and psychiatric diagnoses. Bergen: Department of Psychosocial Science, Faculty of Psychology, University of Bergen.

[bibr32-10870547221105063] Heradstveit O. Skogen J. C. Hetland J. Stewart R. Hysing M. (2019). Psychiatric diagnoses differ considerably in their associations with alcohol/drug-related problems among adolescents. A Norwegian population-based survey linked with national patient registry data. Frontiers in Psychology, 10, 1003. 10.3389/fpsyg.2019.01003 PMC6517475 31133937

[bibr33-10870547221105063] Hogue A. Henderson C. E. Ozechowski T. J. Robbins M. S. (2014). Evidence base on outpatient behavioral treatments for adolescent substance use: Updates and recommendations 2007-2013. Journal of Clinical Child & Adolescent Psychology, 43(5), 695–720. 24926870 10.1080/15374416.2014.915550

[bibr34-10870547221105063] Huss M. Hölling H. Kurth B. M. Schlack R. (2008). How often are German children and adolescents diagnosed with ADHD? Prevalence based on the judgment of health care professionals: Results of the German health and examination survey (KiGGS). European Child & Adolescent Psychiatry, 17 Suppl 1(1), 52–58. 19132304 10.1007/s00787-008-1006-z

[bibr35-10870547221105063] Hysing M. Heradstveit O. Harvey A. G. Nilsen S. A. Bøe T. Sivertsen B. (2020). Sleep problems among adolescents within child and adolescent mental health services. An epidemiological study with registry linkage. European Child & Adolescent Psychiatry, 31, 121–131. 33159591 10.1007/s00787-020-01676-4 PMC8816738

[bibr36-10870547221105063] Indergård P. Fuglset A. Krogh F. Urfjell B. (2019). Aktivitetsdata for psykisk helsevern for barn og unge 2018: Norsk pasientregister [Data on activity in specialised child and adolescent mental health services 2018: Norwegian Patient Registry]. Trondheim: Health Ministry of Norway.

[bibr37-10870547221105063] Keyes K. M. Hatzenbuehler M. L. Hasin D. S. (2011). Stressful life experiences, alcohol consumption, and alcohol use disorders: The epidemiologic evidence for four main types of stressors. Psychopharmacology, 218(1), 1–17. 21373787 10.1007/s00213-011-2236-1 PMC3755727

[bibr38-10870547221105063] Knight J. R. Sherritt L. Shrier L. A. Harris S. K. Chang G. (2002). Validity of the CRAFFT substance abuse screening test among adolescent clinic patients. Archives of Pediatrics & Adolescent Medicine, 156(6), 607–614. 12038895 10.1001/archpedi.156.6.607

[bibr39-10870547221105063] Lee S. S. Humphreys K. L. Flory K. Liu R. Glass K. (2011). Prospective association of childhood attention-deficit/hyperactivity disorder (ADHD) and substance use and abuse/dependence: A meta-analytic review. Clinical Psychology Review, 31(3), 328–341. 21382538 10.1016/j.cpr.2011.01.006 PMC3180912

[bibr40-10870547221105063] Lucas C. P. Zhang H. Fisher P. W. Shaffer D. Regier D. A. Narrow W. E. Bourdon K. Dulcan M. K. Canino G. Rubio-stipec M. Lahey B. B. Friman P. (2001). The DISC predictive scales (DPS): Efficiently screening for diagnoses. Journal of the American Academy of Child and Adolescent Psychiatry, 40(4), 443–449. 11314570 10.1097/00004583-200104000-00013

[bibr41-10870547221105063] Melhem N. M. Walker M. Moritz G. Brent D. A. (2008). Antecedents and sequelae of sudden parental death in offspring and surviving caregivers. Archives of Pediatrics & Adolescent Medicine, 162(5), 403–410. 18458185 10.1001/archpedi.162.5.403 PMC2654289

[bibr42-10870547221105063] Mitchison G. M. Njardvik U. (2019). Prevalence and gender differences of ODD, anxiety, and depression in a sample of children with ADHD. Journal of Attention Disorders, 23(11), 1339–1345. 26443719 10.1177/1087054715608442

[bibr43-10870547221105063] Molina B. S. G. Pelham W. E. (2003). Childhood predictors of adolescent substance use in a longitudinal study of children with ADHD. Journal of Abnormal Psychology, 112(3), 497–507. 12943028 10.1037/0021-843x.112.3.497

[bibr44-10870547221105063] Nock M. K. Kazdin A. E. Hiripi E. Kessler R. C. (2006). Prevalence, subtypes, and correlates of DSM-IV conduct disorder in the national comorbidity survey replication. Psychological Medicine, 36(5), 699–710. 16438742 10.1017/S0033291706007082 PMC1925033

[bibr45-10870547221105063] Norén Selinus E. Molero Y. Lichtenstein P. Anckarsäter H. Lundström S. Bottai M. Hellner Gumpert C . (2016). Subthreshold and threshold attention deficit hyperactivity disorder symptoms in childhood: Psychosocial outcomes in adolescence in boys and girls. Acta Psychiatrica Scandinavica, 134(6), 533–545. 27714770 10.1111/acps.12655 PMC5129548

[bibr46-10870547221105063] Pedersen M. U. Thomsen K. R. Heradstveit O. Skogen J. C. Hesse M. Jones S. (2018). Externalizing behavior problems are related to substance use in adolescents across six samples from Nordic countries. European Child & Adolescent Psychiatry, 27(12), 1551–1561. 29619558 10.1007/s00787-018-1148-6

[bibr47-10870547221105063] Pfiffner L. J. McBurnett K. Rathouz P. J. Judice S. (2005). Family correlates of oppositional and conduct disorders in children with attention deficit/hyperactivity disorder. Journal of Abnormal Child Psychology, 33(5), 551–563. 16195950 10.1007/s10802-005-6737-4

[bibr48-10870547221105063] Rydell A.-M. (2010). Family factors and children’s disruptive behaviour: An investigation of links between demographic characteristics, negative life events and symptoms of ODD and ADHD. Social Psychiatry and Psychiatric Epidemiology, 45(2), 233–244. 19412562 10.1007/s00127-009-0060-2

[bibr49-10870547221105063] Scahill L. Schwab-Stone M. (2000). Epidemiology of ADHD in school-age children. Child and Adolescent Psychiatric Clinics of North America, 9(3), 541–555, vii. 10944656

[bibr50-10870547221105063] Serra-Pinheiro M. A. Coutinho E. S. Souza I. S. Pinna C. Fortes D. Araújo C. Szobot C. M. Rohde L. A. Mattos P. (2013). Is ADHD a risk factor independent of conduct disorder for illicit substance use? A meta-analysis and metaregression investigation. Journal of Attention Disorders, 17(6), 459–469. 22344318 10.1177/1087054711435362

[bibr51-10870547221105063] Sihvola E. Rose R. J. Dick D. M. Korhonen T. Pulkkinen L. Raevuori A. Marttunen M. Kaprio J. (2011). Prospective relationships of ADHD symptoms with developing substance use in a population-derived sample. Psychological Medicine, 41(12), 2615–2623. 21733216 10.1017/S0033291711000791 PMC3707933

[bibr52-10870547221105063] Skogen J. C. Bøe T. Knudsen A. K. Hysing M. (2013). Psychometric properties and concurrent validity of the CRAFFT among Norwegian adolescents. Ung@hordaland, a population-based study.. Addictive Behaviors, 38(10), 2500–2505. 23770648 10.1016/j.addbeh.2013.05.002

[bibr53-10870547221105063] StataCorp. (2017). Stata statistical software: Release 15. College Station, TX: StataCorp LLC.

[bibr54-10870547221105063] Szatmari P. Offord D. R. Boyle M. H. (1989). Ontario Child Health Study: Prevalence of attention deficit disorder with hyperactivity. Journal of child psychology and psychiatry, 30(2), 219–230. 2708462 10.1111/j.1469-7610.1989.tb00236.x

[bibr55-10870547221105063] Tarter R. E. Vanyukov M. Kirisci L. Reynolds M. Clark D. B. (2006). Predictors of marijuana use in adolescents before and after licit drug use: Examination of the gateway hypothesis. American Journal of Psychiatry, 163(12), 2134–2140. 17151165 10.1176/ajp.2006.163.12.2134

[bibr56-10870547221105063] WHO. (1992). The ICD-10 classification of mental and behavioural disorders: Clinical descriptions and diagnostic guidelines (vol.1). World Health Organization.

[bibr57-10870547221105063] Willcutt E. G. Pennington B. F. Chhabildas N. A. Friedman M. C. Alexander J. (1999). Psychiatric comorbidity associated with DSM-IV ADHD in a nonreferred sample of twins. Journal of the American Academy of Child and Adolescent Psychiatry, 38(11), 1355–1362. 10560221 10.1097/00004583-199911000-00009

[bibr58-10870547221105063] Wolke D. Waylen A. Samara M. Steer C. Goodman R. Ford T. Lamberts K. (2009). Selective drop-out in longitudinal studies and non-biased prediction of behaviour disorders. The British Journal of Psychiatry, 195(3), 249–256. 19721116 10.1192/bjp.bp.108.053751 PMC2802508

[bibr59-10870547221105063] Yewers T. Hay D. Barton A. (2005). Attention deficit hyperactivity disorder and severity of drug use in a sample of adult male drug users. Australian Psychologist, 40(2), 109–117.

