# Supplementary material for: Substance-Related Problems in Adolescents with ADHD-Diagnoses: The Importance of Self-Reported Conduct Problems
Source: J Atten Disord. 2022 Jun 25;26(14):1857–69. doi: 10.1177/10870547221105063 (PMC9596946; doi:10.1177/10870547221105063)
Supplement: sj-docx-1-jad-10.1177_10870547221105063 – Supplemental material for Substance-Related Problems in Adolescents with ADHD-Diagnoses: The Importance of Self-Reported Conduct Problems [file sj-docx-1-jad-10.1177_10870547221105063.docx]

**Supplementary files**

*Table S1. Rates of substance-related problems across the defined subgroups of ADHD.*

|  | Substance-related problems | | | |
| --- | --- | --- | --- | --- |
|  | 0 indicators  % (95%CI) | 1 indicator  % (95%CI) | 2 indicators  % (95%CI) | 3+ indicators  % (95%CI) |
| **Total (n=6,300)** |  |  |  |  |
| Survey sample (n=6,130) | 70.3 (69.1, 71.5) | 16.6 (15.7, 17.6) | 8.3 (7.6, 9.0) | 4.7 (4.2, 5.3) |
| ADHD only (n=89) | 68.4 (58.0, 78.9) | 19.7 (10.8, 28.7) | 7.9 (1.8, 14.0) | 3.9 (0, 8.3) |
| ADHD+low conduct problems (n=32) | 58.1 (40.7, 75.4) | 22.6 (7.9, 37.3) | 9.7 (0, 20.1) | 9.7 (0, 20.1) |
| ADHD+ high conduct problems (n=49) | 33.3 (19.1, 47.6) | 21.4 (9.0, 33.8) | 16.7 (5.4, 27.9) | 28.6 (14.9, 42.2) |
| **Boys (n=2,831)** |  |  |  |  |
| Survey sample (n=2,739) | 71.8 (70.0, 73.6) | 15.4 (14.0, 16.9) | 7.9 (6.9, 9.0) | 4.8 (4.0, 5.7) |
| ADHD only (n=41) | 76.5 (62.2, 90.7) | 11.8 (0.9, 22.5) | 5.9 (0, 13.8) | 5.9 (0, 13.8) |
| ADHD+low conduct problems (n=19) | 72.2 (51.5, 92.9) | 16.7 (0, 33.9) | 5.6 (0, 16.1) | 5.6 (0, 16.1) |
| ADHD+ high conduct problems (n=32) | 46.4 (28.0, 64.9) | 14.3 (1.3, 27.2) | 17.9 (3.7, 32.0) | 21.4 (6.2, 36.6) |
| **Girls (n=3,469)** |  |  |  |  |
| Survey sample (n=3,391) | 69.1 (67.5, 70.7) | 17.6 (16.3, 18.9) | 8.6 (7.6, 9.6) | 4.6 (3.9, 5.4) |
| ADHD only (n=48) | 61.9 (47.2, 76.6) | 26.2 (12.9, 39.5) | 9.5 (0.6, 18.4) | 2.4 (0, 7.0) |
| ADHD+low conduct problems (n=13) | 38.5 (12.0, 64.9) | 30.8 (5.7, 55.9) | 15.4 (0, 35.0) | 15.4 (0, 35.0) |
| ADHD+ high conduct problems (n=17) | 7.1 (0, 20.6) | 35.7 (10.6, 60.8) | 14.3 (0, 32.6) | 42.9 (16.9, 68.8) |
|  |  |  |  |  |
|  | | | | |
